# *In vivo* protein corona on nanoparticles: does the control of all material parameters orient the biological behavior?

**DOI:** 10.1039/d0na00863j

**Published:** 2021-01-13

**Authors:** Nimisha Singh, Célia Marets, Julien Boudon, Nadine Millot, Lucien Saviot, Lionel Maurizi

**Affiliations:** Laboratoire Interdisciplinaire Carnot de Bourgogne (ICB), UMR 6303 CNRS – Université Bourgogne Franche-Comté BP 47870 Dijon Cedex F-21078 France lucien.saviot@u-bourgogne.fr lionelmaurizi@gmail.com

## Abstract

Nanomaterials have a huge potential in research fields from nanomedicine to medical devices. However, surface modifications of nanoparticles (NPs) and thus of their physicochemical properties failed to predict their biological behavior. This requires investigating the “missing link” at the nano-bio interface. The protein corona (PC), the set of proteins binding to the NPs surface, plays a critical role in particle recognition by the innate immune system. Still, *in vitro* incubation offers a limited understanding of biological interactions and fails to explain the *in vivo* fate. To date, several reports explained the impact of PC *in vitro* but its applications in the clinical field have been very limited. Furthermore, PC is often considered as a biological barrier reducing the targeting efficiency of nano vehicles. But the protein binding can actually be controlled by altering PC both *in vitro* and *in vivo*. Analyzing PC *in vivo* could accordingly provide a deep understanding of its biological effect and speed up the transfer to clinical applications. This review demonstrates the need for clarifications on the effect of PC *in vivo* and the control of its behavior by changing its physicochemical properties. It unfolds the recent *in vivo* developments to understand mechanisms and challenges at the nano-bio interface. Finally, it reports recent advances in the *in vivo* PC to overcome and control the limitations of the *in vitro* PC by employing PC as a boosting resource to prolong the NPs half-life, to improve their formulations and thereby to increase its use for biomedical applications.

## Abstract 2

Recent advances in understanding and controlling the *in vivo* protein corona on nanoparticles to optimize the biological response.

## Graphical abstract

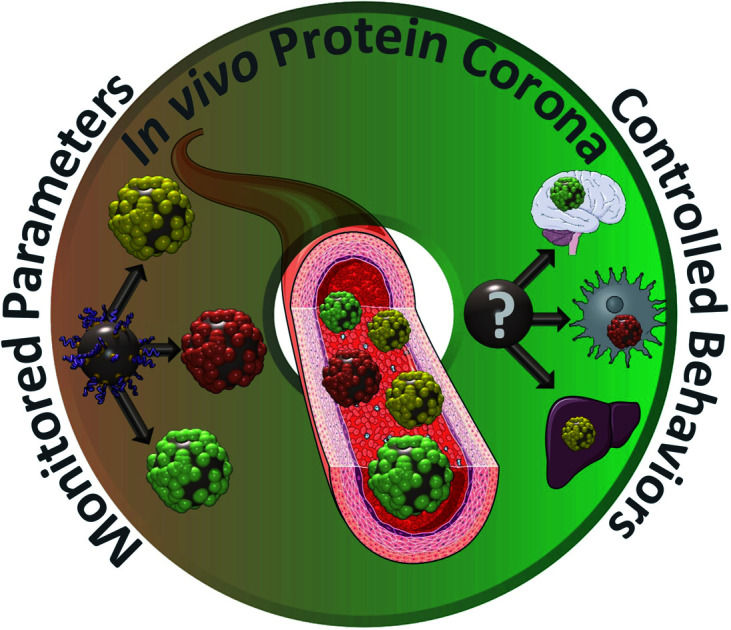

## Introduction

The idea of using nanomaterials for diagnoses and treating deadly diseases has driven biomedical research for decades. Nanoparticles (NPs) can be used in many biological and medical fields as diagnostics probes,^1–4^ drug delivery vectors^5,6^ or for other therapeutic purposes.^7–10^ In all these applications, the surface of NPs have to be functionalized in order to improve stability, biocompatibility or targeting efficiency.^11–13^ Surface chemistry is an inherent parameter in the set-up of innovative nanohybrids. Small drug molecules often suffer from poor pharmacokinetics. They exhibit rapid clearance and fail to reach the targeted sites *in vivo.* Consequently, severe side effects may result from the expected therapeutic benefits. In addition, the *in vitro* efficacy of drugs often translates poorly in a clinical setting.^14^ Using NPs can help homogenize and improve the efficacy of therapeutic molecules by increasing their retention, circulation times and targeting. Engineering NPs allow optimizing different parameters: charge, size, shape and surface chemistry including the nature of the nanoparticle itself (metallic or organic) or the molecules used to functionalize them (polymers with different chemical groups and lengths, antibody *etc.*). These functionalizations influence the NPs' biological behaviors. For example, it was reported that the surface chemistry of NPs controls their *in vivo* biodistribution due to the type of corona formed round them as observed after tail vein injection into mice. While there are significant differences in the behavior depending on the type of NPs, all the investigated NPs, with hydrodynamic sizes higher than 30 nm, are predominantly located in the liver and spleen as reported by Xu *et al.*^15^ An important point in designing a successful nanomedical tool is to understand the biological interactions with all these diverse engineered NPs with optimized size, shapes, charge *etc.* on the surface. NPs' charges also modify the uptake or the biodistribution of NPs.^16–19^ However, such observations are not only the result of NPs charges but also of the interaction occurring at the interface between NPs and biological fluids.

**Fig. 1:**
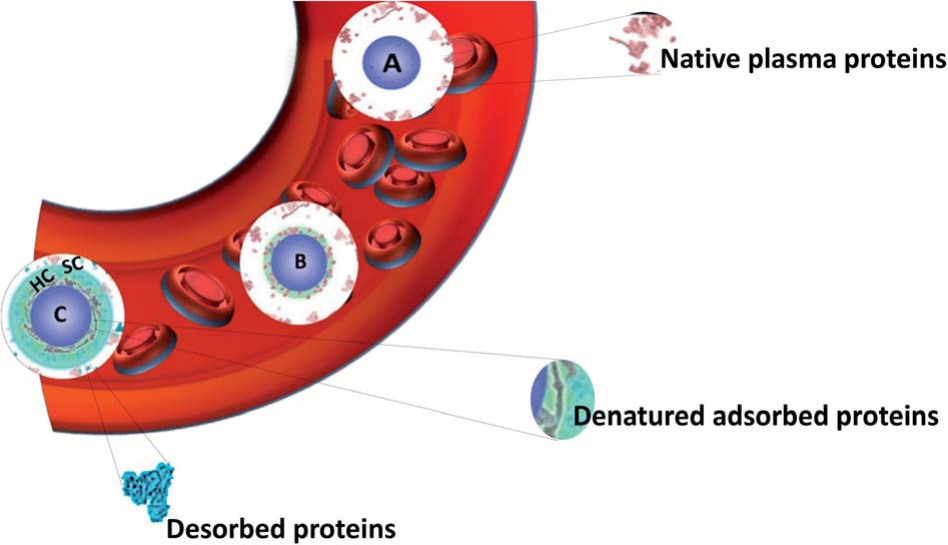
Schematic view of the formation of the hard protein corona around a nanoparticle in blood plasma. Adapted from ref. 21 Copyright© (2016) with permission from Elsevier. (A) Nanoparticle entering into the plasma, (B) smaller proteins adsorb first and some are denatured forming a soft corona (SC), and (C) larger proteins replacing the smaller ones (Vroman effect) followed by denaturation to achieve irreversible adsorption forming HC.

In fact, after introduction into a biological fluid, the surfaces of NPs are immediately surrounded by biomolecules such as proteins, lipids, sugars and nucleic acids. It is then difficult to predict the nanosystem surfaces once it is exposed to the biological medium as NPs evolve differently in biological media. Molecules interact through different forces with NPs (London dispersion forces, Coulomb forces, van der Waals forces, hydrogen bonding and hydrophobic effects) causing the formation of a corona. Among these biomolecules, proteins turn out to play a significant role in the formation of the so-called protein corona (PC) as shown by proteomics studies. This term was introduced in 2007 by Cedervall *et al.*^20^ In this study, they also introduced the terms “hard corona” (HC) and “soft corona” (SC). SC results from proteins involved in temporary low affinity interactions while HC largely results from permanent high affinity interactions as schematized in Fig. 1.^21^ Proteins having higher affinities and resulting in HC interact first with the NPs followed by the ones forming SC. In fact, when interactions are observed as a function of time, SC results from short time interactions while HC is made of proteins which bind to the NPs for several hours.^22^ Walkey *et al.*^23^ suggested that analyzing the HC proteins should be more relevant than analyzing the SC proteins to predict the biological responses of NPs. It was indeed reported for many nanomaterial systems that the HC contribution dominates the biological responses such as, for example, with CdSe/ZnS quantum dots in human blood serum^24^ or with the analysis of hemolysis using graphene oxide functionalized with d-mannose.^25^ HC is now considered to be the most important corona to analyze.^26^ As a result, it is often confused with the classical PC. Therefore, in all the studies summarized in this review and the literature, PC refers mostly to HC. For ease of understanding, the different forms of corona mentioned in this review including bio corona, SC, HC, preformed corona, *in vitro*, *in vivo* corona, synthetic corona, polymer corona *etc.* will be referred to as PC.

The formation of PC is a spontaneous and competitive process. It is influenced by the surface properties of NPs (size,^27^ charge,^28^ shape,^29^ chemistry^30–32^*etc.*), environmental parameters such as the nature of the biological medium^33^ (protein source and concentration), as well as the exposure time.^34^ It is nowadays established that PC, in turn, also control the NPs' biological identity and behavior. For instance, positively charged NPs are usually recognized by opsonins which can result in their faster elimination from the mononuclear phagocytic system (MPS).^30^ Thus, negatively charged NPs leading to negative zeta potentials (in the range of (−50; −30) mV) are more prone to avoid opsonization in physiological conditions.^35^ Ideally, intravenous (i.v.) administered NPs undergo MPS which recognizes the foreign substances by adsorbing specific serum proteins *via* opsonization. It is reported that the presence of opsonins (blood proteins, such as immunoglobulins (Ig) and complement factors) in the corona facilitates the NPs cellular uptake through the opsonin-cognate receptors expressed on the phagocytic surface.^36,37^*In vitro*, adsorbed protein layers reportedly also influence the cellular uptake^38^ and trafficking,^39^ whereas *in vivo* preferential binding of proteins may affect the particle distribution.^31^ These are some observations suggesting that several *in vitro* and *in vivo* evaluations do not correlate. However, the underlying question for understanding the influence of the NP–protein interactions on the biological response to NPs *in vitro* and *in vivo* remains unanswered.

Several parameters influence the *in vitro* incubation of NPs. They provide an insight on the biological interaction with NPs. But up to now they have failed to explain the fate^40,41^ of NPs *in vivo*. Not enough publications highlight the impact of PC on the *in vivo* behavior of NPs even if this limits their potential application in biomedicine. The main reason for this situation is that *in vivo* evaluations are technically very complicated and expensive. In this review, we will be exploring various aspects of PC *in vivo* and we will present and discuss the last advances in this field. After a short summary of the key parameters responsible for the formation of the PC obtained in *in vitro* studies, we will unravel the PC formation and how it can be controlled in designing nanomaterials for targeted *in vivo* applications. Then, we will focus on studies that actually investigated *in vivo* the PC formation on NPs. We will discuss the key parameters influencing PC *in vivo* and the differences observed between *in vitro* and *in vivo* analyses. We will finally discuss some open questions and inherent problems of this recent research field, the limitation of which is crucial to overcome in designing nanotools for biomedical applications.

## Parameters influencing the protein corona

Before focusing on the studies dedicated to the control and the understanding of PC *in vivo*, it is important to present succinctly the main advances obtained with *in vitro* measurements and studies. The PC formation on NPs' surfaces has been found to be highly dependent on experimental parameters (NPs composition, shape, size, surface charge, roughness, protein type, concentrations, pH and ionic strength of the biological media). These can be used to tune the composition or minimize the formation of PC.^42–44^ The parameters influencing PC can be divided into two categories: environmental parameters and material parameters.

## Environmental parameters influencing the PC formation

**Fig. 2:**
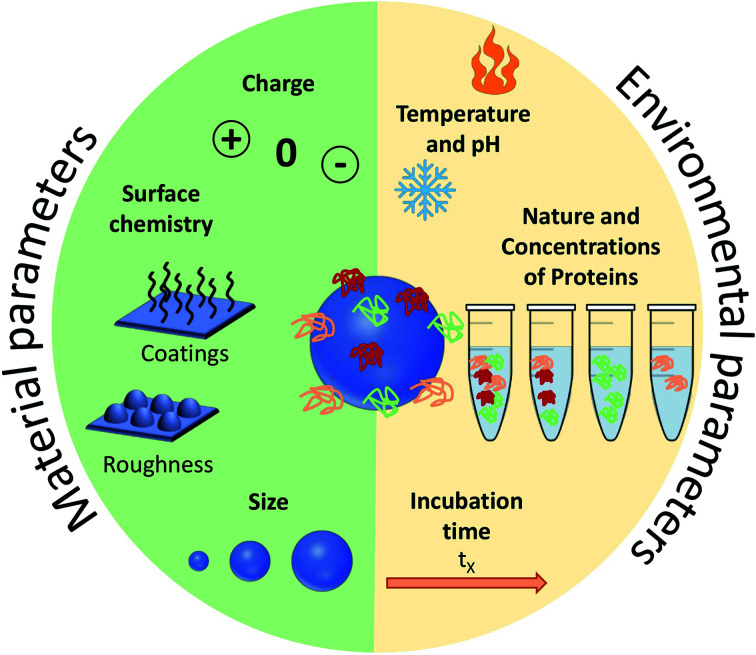
Summary of the main parameters influencing the formation of protein corona (PC). These parameters are divided into two: the ones related to the environment and those related to the NPs.

The adsorbed proteins forming PC are known to play a key role during the interaction of NPs with cells. Several environmental parameters (Fig. 2) affects the formation of PC *in vitro* particularly the medium surrounding the NPs and the exposure conditions. The biological fluid or the medium is composed of different proteins which are known to control the cellular uptake of NPs. This was explained by Tekie *et al.*^45^ for the uptake of chitosan and carboxylmethyl dextran complexes (MCF7 cell line). Fetal Bovine Serum (FBS) enhances the uptake due to the presence of proteins in the serum such as alpha-trypsin inhibitor chains and lipoproteins which increase the cell function. Similarly, proteins concentration and exposure time contribute equally to the protein adsorption on NPs. Controlling the PC formation is undoubtedly a challenging task but several results highlight that altering the biological parameters along with the physicochemical properties of NPs enable to forecast the corona formation. As analyzed by Partikel *et al.*^46^, proteins adsorption on poly(lactide-*co*-glycolic acid) (PLGA) NPs depends on the serum type and concentration. In addition, human serum was shown to significantly modify the corona composition resulting in a concentration-dependent desorption of abundant proteins along with the adsorption of high affinity proteins with lower abundance. In addition, time-dependent cell interaction both in the absence and the presence of a preformed corona showed a significant influence on a human liver cancer cell line (HepG2) in which the presence of corona increases the cell interaction compared to bare NPs which results in a higher uptake of NPs. Temperature and pH are also important factors in the interaction of NPs with proteins. Galdino *et al.*^47^ explained that pH influences the protein adsorption in the Bovine Serum Albumin (BSA) and SiO_2_ NPs system. They concluded that enthalpy controls the interaction along with an electrostatic contribution that can be altered by changing pH. Gorshkov *et al.*^48^ further explained that by varying pH and temperature in the human blood plasma and a silver NPs system, different tertiary protein structures and charge localizations are observed. PC formation is a dynamic process involving complex interactions. These interactions are highly dependent on the biological environment but the NPs surface chemistry and properties must not be ignored. Thus, a careful evaluation of both the nano-bio surfaces and interactions can further help investigate the manifold behaviors of NPs.

Many external parameters influence the formation of PC on NPs. With the aim of controlling PC for *in vivo* applications, most of these parameters would be difficult to modify, because living bodies are complex organisms with varying pH, temperature, concentration and nature of proteins *etc*. Solutions to control the PC formation might therefore mainly come from the NPs' surface itself that also influences proteins' adsorption.

## Material parameters influencing PC

In addition to the biological fluids' properties, the particulate nature of NPs dictates a preferential interaction on biointerfaces depending on the physiochemical properties of their surfaces as illustrated in Fig. 2. Size, charge and more generally the chemistry of NPs' surfaces influence the amount and composition of adsorbed proteins.

### Impact of the size of NPs

The surface of NPs strongly attracts blood proteins. The binding constant and the number of binding sites are highly dependent on the NPs dimensions because of the surface curvature. 37% of all the proteins identified in the corona show significant variations in their binding behavior when varying the size of NPs.^49^ This strongly suggests that the size directly affects the nature of PC. In mouse serum not only a larger number of proteins binds bigger NPs but also a more complex PC is formed.^27^ However, in some cases, the reverse phenomenon is observed. This is the case for TiO_2_ NPs with pepsin where smaller sizes result in a larger number of proteins on the surface.^50^ Polystyrene (PS) NPs with Human Serum Albumin (HSA) follow this trend too.^51^ In addition, reports in the literature also show that the size of NPs affects the protein abundance in PC but also changes the PC composition affecting its biological fate^52^ as explained by Zhang *et al.*^53^ with different sizes of silica NPs towards the protein composition of PC. The protein pattern observed for the same mass and different sizes of NPs suggests that increasing the NPs size decreases the number of adsorbed proteins of either the interfacial ones (proteins between the NPs and corona phases) or those from HC, which is consistent with the decrease of the NPs' surface area.

Ho *et al.*^54^ studied the impact of PC on the cellular uptake in human umbilical vein endothelial cells (HUVAC) of PS NPs and PEG-functionalized PS NPs of different sizes. PS NPs and PS–PEG NPs of 20 nm and 40 nm showed no uptake difference. However, for larger PS NPs (100 nm and 200 nm), PC significantly increased the uptake by 10 times compared to PEG-functionalized NPs. On the other hand, PEG-functionalized PS NPs follow the opposite trend. Although the less statistical difference was reduced, the uptake was greater for smaller NPs (20 nm and 40 nm) by 1.3 times compared to PS NPs. Less is known about the uptake behavior in the presence of PEGylated NPs of different sizes. Similar studies were performed on corona-coated Au NPs. Larger sizes favored an higher uptake in HepG2 cells.^55^ This increase is possibly due to the difference in composition and conformation of the serum proteins forming PC. Proteins bound to larger NPs tend to undergo conformational changes to adjust in larger surface area and surface curvature.^34^ Similar results were obtained by Binnemars-Postma *et al.*^56^ when analyzing the uptake of silica NPs by M1 and M2 macrophages in the presence of serum. Remarkably, 500 nm and 1000 nm NPs had a greater uptake in Monocytic human THP-1 cells than the 200 nm ones. This suggests that the adsorption of serum proteins on larger particles favors the uptake, that the PC which is formed is enriched with complement factors and that IgG likely governs the higher uptake of larger NPs.

Conclusively, NPs surface curvature strongly affects the protein adsorption as protein-binding affinities are different for the bulk material and NPs surface. Thus, corona formed on NPs made of the same material differ in composition.^57^ Undoubtedly, PC formation is a continuous process with changes in PC composition with time due to the motion of already adsorbed proteins which may be replaced by other proteins having stronger binding affinities until the process reaches an equilibrium which is known as the “Vroman effect”.^58^ On the other hand, in terms of sorting preferred biological medium, it depends on the choice of molecule to be selected in order to form PC which depends on the orientation on the surface and the degree of unfolding which permits various interactions based on the charge, hydrophobicity *etc.*

### Impact of the charge of NPs

Electrostatic forces play an important role in the adsorption of proteins. It was observed that low surface charges result in fewer adsorbed proteins with distinctively slower opsonization than strongly charged particle surfaces.^59^ This suggests that zwitterionic coatings (amino acids, sulfobetaine, phosphorylcholine, poly(acrylic acid), and poly(maleic anhydride-*alt*-1-alkene) derivatives) can lead to a greater reduction in the adsorption of proteins.^60,61^ For example, sulfobetaine headgroup can be designed with controlled hydrophobicity preventing the adsorbtion of proteins and the formation of PC when observed with human serum and MCF-7 (breast adenocarcinoma) cell line. Indeed, Debayle *et al.*^62^ compared sulfobetaine with other zwitterionic polymers (phosporylcholine and carboxybetaine). A complete absence of PC was observed with sulfobetaine. Other polymers showed reversible adsorption and aggregation. Additionally, positively charged NPs form a thicker PC than negatively charged ones as exhibited by paclitaxel loaded poly(ε-caprolactone) on MCF-7 cell line and HSA medium.^63,64^ Liposomes, which can be considered as organic NPs, depict the same behavior with charged surfaces adsorbing more proteins than neutral ones. Additionally, liposomes made of anionic or cationic lipids preferentially adsorb plasma proteins with isoelectric point (IEP), IEP > pH 5.5 or IEP < pH 5.5 respectively.^65^ The chirality of the functionalized group may also be responsible for different protein binding behavior as observed by Qu *et al.*^66^ with InP@ZnS quantum dots. The adsorption of proteins (HSA) differs with the chirality (d- and l-penicillamine) of the functionalized group, impacting the binding affinity and conformation states. This leads to different biological interactions and protein exchange.

Different charge modifications on the same kind of NPs often result in different structural conformations of proteins. For PS NPs, either a protein conformation change is observed or it remains unaffected, with NH_2_ or COOH surface functionalization respectively.^28^ In fact, one type of proteins when adsorbed shows different secondary structure depending on the chemical group charges. Different epitopes can be exposed thus influencing the interaction of NP–PC complexes.^28^ This in turn influences the various paths for cell internalization resulting in different uptake behavior. Indeed, particle uptake is often triggered by phagocytosis where NPs interact with the responsible receptors on the cell surface. Various functionalized PS NPs were also studied to understand the intracellular fate of PC. A larger number of proteins was carried on the aminated surface and degraded within the lysosomes.^67^ PS–NH_2_ NPs thus showed a 5 times more internalized PC (following endocytosis, where the NPs are entrapped by the cell membrane and drawn into the cell) than their COOH counterparts as measured by flow cytometry. This consequently gives rise to a different uptake process on A549 cells (adenocarcinomic human alveolar basal epithelial cells) with an increased exchange rate of corona in contact with the cellular recognition machinery. Once internalized, most NPs follow the lysosomal pathways. Additionally, amino groups on the surface are a predominant factor in the formation of PC and have a subsequent impact on the cellular uptake, which is controlled by its amine type, location and density. Similar experiments were performed with two cell lines (A549 and J774A.1, mouse monocyte macrophage cell line) to see the effect of the amine bulkiness *in vitro*. It was observed that primary amino groups lead to an increased NP–cell interaction compared to secondary and tertiary amino groups followed by an enhanced uptake.^68^ This is due to the amine bulkiness, which promotes the formation of salt bridges and its hydrophobicity which alters the adsorbed PC.

### Impact of the surface chemistry of NPs

Surface chemistry is also an important parameter allowing to orient the affinities and type of proteins that adsorb on NPs. Chen *et al.*^69^ recently found that the surface chemistry of lipid NPs (LNPs) is correlated to the PC composition and suggested a potential application to targeted delivery. LNPs with different polyethylene glycol (PEG) chain length showed significant differences in cellular delivery and transfection in HepG2 cells in the presence and absence of FBS. Cai *et al.*^70^ further found out that surface chemistry has a more pronounced effect on the PC composition than the surface charge. Their study concerned gold nanorods functionalized with different ligands to analyze the cellular pathways (human leukemia cell line, THP-1) followed by subsequent mononuclear phagocytic system recognition behavior. These studies further help in determining the long-term stability of the NPs, their biological transport and fate when using selective surface ligand. In another study, Sakulkhu *et al.*^71^ investigated the role of the chemical coatings on the surface of iron oxide and silica NPs. They demonstrated that superparamagnetic iron oxide NPs (SPIONs) coated with polyvinyl alcohol (PVA) polymers with different lengths and chemical groups drastically change their PC composition after incubation in FBS. For instance, only five proteins were found on the surface of SPIONs coated with carboxy–PVA (with COOH groups) when 54 different proteins were found for amino-PVA-coated SPIONs (with NH_2_ groups). However, interactions can also originate from the charges of SPIONs (negative for PVA–COOH and positive for PVA–NH_2_). The chemistry of polymers was also a parameter in the proteins' adsorption. In a different approach, P. Chandran *et al.*^72^ explained that larger charged Au NPs possess a greater protein binding when functionalized with lipoic acid (LA) compared to NPs functionalized with branched polyethyleneimine (BPEI) despite being strongly cationic. It further confirms the size- and surface chemistry-dependent uptake in HUVAC of corona-bound Au NPs.

### Impact of the surface roughness of NPs

Surface chemistry modifies various properties (hydrophobicity and charge) that consequently changes the PC composition. However, surface roughness cannot be neglected when analyzing the PC formation around NPs. Since higher surface roughness causes greater protein adsorption,^73^ it results in lower uptake as observed for polymer-coated silica NPs on HeLa cells.^74^ On the contrary, Piloni *et al.*^75^ analyzed the surface roughness on three cell lines namely macrophages (RAW264.7), breast cancer cells (MDA-MB-231) and fibroblasts (Hs27). They observed that rough surfaces reduce the PC formation supporting non-specific binding compared to protein-coated smooth surface particles with a thick PC layer. The latter show a higher uptake on all the observed cell lines.

Different formulations of liposomes were recently studied by Foteini *et al.*^76^ in FBS medium. They observed packing defects due to the presence of long phospholipid chains. This results in exposing hydrophobic domains on the surface of the bilayer thereby enhancing the interaction between proteins and fatty acyl chains. However, stability in the medium and uptake were found to be concentration-dependent.

## Controlling PC *in vitro* for *in vivo* applications

Many studies try to tune the PC *in vitro* in order to control the *in vivo* behaviors of NPs. The environmental parameters presented in the previous part are difficult or even impossible to control *in vivo* except perhaps for the incubation time (more explanations in the next part). Thus, it is more interesting to focus on engineering the NPs surface in order to tailor the formation of PC *via* different approaches: (i) controlling the surface chemistry of NPs or (ii) precoating NPs *in vitro* with proteins to have a controlled PC for optimized biological interactions.

### Control of the surface chemistry to tune PC for *in vivo* applications

Surface chemistry plays a very important role as discussed in the previous part in orienting the PC composition on NPs. Various physiochemical parameters control PC inside the body. For example, by studying various NP sizes, it stands out that highly negative charges increase the circulation time. This is in turn directly influenced by the extent of the PC formed around NPs. PC thus shows the potential to alter the synthetic identities of NPs and affect their interaction on different encounters with *in vivo* biological entities followed by their body retention and excretion time. As also explained by Kenry *et al.*,^77^ the surface charge controls the biodistribution of polymeric NPs and negatively charged NPs show a longer circulation time with minimal macrophage uptake compared to positively charged ones. This further enables lesion penetration and the accumulation of NPs at the targeted sites for theranostic application. The concept was further confirmed by Landgraf *et al.*^78^, when Au–Fe_3_O_4_–SiO_2_–PEG janus particles were shown to have more PC around them after incubation when compared to Fe_3_O_4_–SiO_2_–PEG NPs. Additionally, several attempts were made for which various functional groups like phycocyanin,^79^ methyl phosphonate, PEG^80^*etc.*, are functionalized and adjusted on the NPs with the potential to preform a PC structure through non-covalent interface interactions. This preformed PC improves dispersion in water and inhibits the plasma protein adsorption thereby improving biocompatibility *in vivo.* This in turn influences the phototherapeutic efficacy as observed in tumor bearing mice suggesting feasible synergistic photodynamic therapy (PDT)/photothermal therapy (PTT) nanoplatform for the treatment of cancer.^79^ Tumor-bearing mice were prepared for the experiment by subcutaneous injection of a suspension of 51064T1 cells. Chen *et al.*^69^ further showed that PC can be manipulated by varying the surface charges. By changing components in lipid NPs, it is possible to tune the surface charge. The authors showed that introducing positively charge lipids results in shifting the PC pattern from apolipoprotein (Apo)-rich to vitronectin-rich. It results in less tumor accumulation in HepG2 tumor bearing mice, while neutral charged NPs have the best tumor accumulation. These changes had a great impact on cell transfection, *in vivo* biodistribution and tumor specific delivery efficiency.

**Fig. 3:**
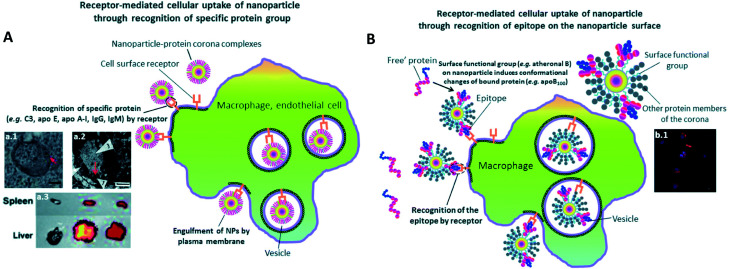
The presence of PC induces receptor-mediated cellular uptake of NPs. (A) Recognition of specific bound protein groups by the cell surface receptors results in a receptor-mediated cellular uptake of NPs: (a1) receptor-mediated uptake of C3-opsonised NPs by macrophage (scale bar = 100 nm). Reprinted with permission from ref. 85 Copyright© 2012, (a2) interaction of bound Apo E with cell surface receptors such as LDLR, VLDLR and apo ER-2 facilitates the uptake of NPs into blood endothelial cells of the brain (scale bar = 1 μm). Reprinted from ref. 87, Copyright© (1995) with permission from Elsevier, (a3) a higher extent of opsonization of NPs by IgM relative to IgG results in a higher liver to spleen particle accumulation ratio. Reprinted with permission from ref. 86 Copyright© 2013 (B) conformational changes of bound protein may result in presentation of a new epitope (antigenic determinant) on the NP's surface. Recognition of the epitope by cell surface receptor facilitates the cellular uptake of NPs: (b1) uptake of quantum dots by macrophage *via* epitope recognition by the cell surface receptor (scale bar = 10 μm). The epitope originates from conformational changes of apo B100 upon binding to atheronal B-modified quantum dots. Reprinted with permission from ref. 88 Copyright© 2012 (A and B) are reproduced from ref. 84 published by the Royal Society of Chemistry.

Another interesting approach^81^ showed the successful demonstration of ganglioside GM3-mediated antigen presenting cells (APC) targeting *in vivo*. Enveloped virus inspired artificial virus NPs (AVN) were prepared offering a dual mode treatment combining the self-assembled membrane as a matrix for bioactive lipids and a protein-repellent coating with the unique properties of the NP core. Despite the formation of a PC, GM3 embedded in the AVN membrane remained accessible to CD169 receptor binding and achieved a selective homing to the peripheral regions of lymph nodes that are enriched in CD169 + APCs *in vivo*. Similar studies reported that forming a PC can alter the physiochemical properties thereby affecting the specific functionality. For example, legumain-responsive Au NPs after incubation in mouse plasma proteins kept the legumain-responsiveness *in vitro.* Ruan *et al.*^82^ studied a drug delivery system, composed of two types of NPs. One was Ala–Ala–Asn–Cys–Lys–polyethylene glycol–thiol (AK–PEG–SH) modified citrate-stable AuNPs coloaded with pH-sensitive DOX and pH-sensitive hydroxychloroquine (HCQ) prodrug (D&H–A–AK) through the “SH–Au” chelation. The other was 2-cyano-6-amino-benzothiazole-polyethyleneglycol-thiol (CABT–PEG–SH) modified AuNPs coloaded with DOX and HCQ (D&H–A–CABT). The system of those two NPs was named “D&H–A–A&C”. The combined therapy on Au NPs were shown to target the glioma sites *in vivo* which even on forming PC still possessed the legumain responsiveness. These approaches are very effective in designing the personalized combination therapeutic regimen for glioma patients, who are patients affected by a type of tumor in the brain and spinal cord. PC formation further helps in developing atherosclerotic vaccines as studied on mice by Benne *et al.*^83^ Liposomes containing the anionic phospholipid 1,2-distearoyl-*sn-glycero*-3-phosphoglycerol (DSPG) facilitate the PC formation *via* scavenger receptors (SR). This results in higher uptake and induces a high number of antigen specific Treg responses (regulatory T cells) compared to the serum free condition after a single injection of DSPG liposomes. Similarly, PC also induces receptor-mediated cellular uptake controlled by surface functional groups as also explained in Fig. 3.^84^ PEG grafting on Au NPs decreases the adsorption of complement protein resulting in lowering of the macrophage uptake (Fig. 3(a1)).^85^ On the contrary, PEG on carbon nanotubes supports higher adsorption of IgM which results in lower ratio of spleen *versus* liver accumulation of NPs (Fig. 3(a3)).^86^ Additionally, to promote preferential binding of selective protein like Apo E, surfactant was grafted on the NPs acting as anchor for Apo E thereby promoting endocytosis^87^ (Fig. 3(a2)). Another example of preferential binding of Apo B to CdSe/ZnS quantum dots resulted in presenting a new epitope (antigenic determinant) giving receptor-mediated uptake of NPs by macrophages (Fig. 3(b1)).^88^

In a recent study, Wu *et al.*^89^ reported that polyphenylene dendrimers (PPD)-controlled amphiphilic surfaces patches showed the potential for forming PC that enabled their interaction with human adenovirus 5 (Ad5) *in vivo* distribution. Ad5 results in the accumulation of viral particles in the liver after intravenous administration and then transduction takes place. *In vivo* studies showed that PC had reduced by about 40% the Ad5-mediated transduction marked by EGFP expression in the liver. Surprisingly, it also increased the transduction in the heart by more than 40% when compared to naked Ad5. These approaches in which PC can manipulate and reengineer the Ad5 biodistribution, prove their potential in regulating the trafficking and cell uptake of viruses *in vivo*, stated as the holy grail of gene therapy.

**Fig. 4:**
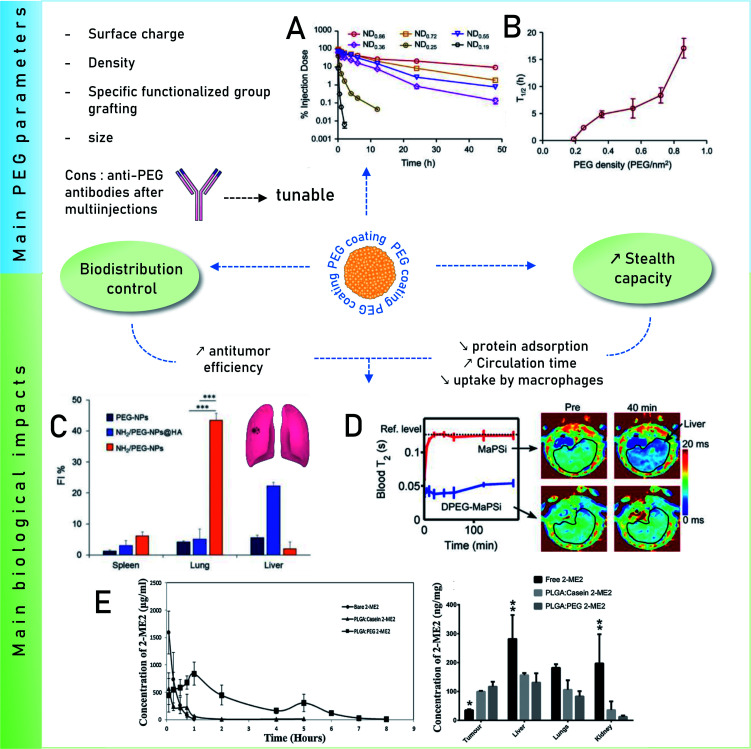
Main impacts on biological behaviors of PEG coatings. Influence of surface PEG densities on the pharmacokinetics parameters Reprinted from ref. 93, Copyright© (2015) with permission from Elsevier: (A) concentration as a function of time for NPs with varying PEG densities in mice plasma after i.v. administration and (B) their terminal half-time (*t*_1/2_). (C) Quantitative *in vivo* organ distribution of intravenously-injected NPs. Mice were i.v. injected with 3 × 10^5^ B16F10 murine melanoma cells *via* the tail vein, treated with NPs in saline after 1 week (lung colonization model), Reprinted from ref. 100, Copyright© (2018) with permission from Elsevier. (D) Relaxation rates of MRI-traceable superparamagnetic mesoporous silica NPs (MaPSi) and DualPEG (DPEG–MaPSi). Map of a rat liver before and 40 min after the 2 mL, 0.5 mg mL^−1^ injections of MaPSi or DPEG-MaPSi NPs. The black line delimits the liver. Reprinted (adapted) with permission from ref. 92. Copyright© 2020 American Chemical Society. (E) *In vivo* pharmacokinetics of bare 2-ME2 and nano formulations and their *in vivo* tumor accumulation and biodistribution. Reprinted from ref. 91, Copyright© (2017) with permission from Elsevier.

One recurring goal is to gain stealth capacity in order to allow NPs to reach their respective medical target by increasing the circulating time in blood. Macrophages (Kupffer cells, or macrophages of the liver) potentially remove unprotected NPs from the bloodstream within seconds after i.v. administration, inhibiting targeted drug delivery. These macrophages on the other hand recognize specific opsonin proteins instead of directly identifying the NPs. Thus, several methods have been employed to camouflage the NPs allowing them to bypass MPS recognition thereby increasing their blood circulation life.^90^ PEG is thus one of the most common coatings used to avoid recognition by the opsonins, which has been broadly explored. It is still investigated owing to its advantageous pharmacokinetic properties, which could be due to its influence on protein adsorption. It has been shown several times that a PEG coating reduces protein adsorption compared to other coatings^91^ or bare NPs. For instance, Nissinen *et al.*^92^ added a DPEG (DualPEG) coating using two kind of PEG simultaneously on mesoporous silicon NPs (PSi-NPs). Such coatings affect the PC composition. Indeed, a smaller number of proteins were adsorbed on DPEG-NPs compared to bare-NPs, especially less liver and immune response associated ones but more phagocytosis inhibitions proteins. Thus, DPEG-coated NPs resulted in a significantly increased circulating time. Tuning the PEG density also controls the protein adsorption as demonstrated by Du *et al.*^93^ and illustrated in Fig. 4. A high surface density reduces the protein adsorption significantly and the uptakes by macrophages, enhancing the antitumor efficiency of the NPs carrying docetaxel *in vivo*. In addition, modified PEG helped obtain a selective corona on NPs. Li *et al.*^94^ showed the interest of selective adsorption of apolipoprotein E (Apo-E), known as endogenous lipid-based transporting protein, for tumor-homing chemotherapy. Dihydroartemisinin (DHA)-decorated NP surfaces were engineered to anchor Apo-E. Then PLGA–PEG_2000_–DHA (PPD) NPs have an Apo-E-enriched corona prolonging the NPs blood circulation thereby facilitating their accumulation in tumor cells by the passive enhanced permeability and retention (EPR) effect. On observing the anti-tumor activity on 4T1 tumor harboring Balb/c mice, it proved a delayed tumor growth performance and triggered significant tumor cell apoptosis with no change in body weight, organ index or haematological parameters. Similarly, *in situ* albumin-enriched corona was explored by the same group.^95^ Maleimide-coated NPs were prepared that preferentially bind endogenous albumin in the corona allowing NPs to stealth and tumor homing ability. These *in situ* approaches have improved delivering efficient chemotherapy with minimum off target toxicities.

Remarkably, for iron oxide nanoparticles (IONPs) coated with glucose or PEG, it was observed^96^ that both surface coatings adsorbed a similar number of proteins *in vitro* but there was a clear difference in the PC composition which was correlated to the NP biodistribution *in vivo*. This results in slower degradation of the glucose coating *in vitro* than *in vivo* where an accelerated biodegradation and clearance were observed for PEG coating in both liver and spleen. The reason for faster *in vivo* degradation lies in the composition of the PC. Glucose-functionalized IONPs had opsonins while PEG was enriched with albumin that degrades faster PEG is known to inhibit the formation of PC. However, it further raised a concern over using PEG for prolonged circulation time due to the finding of anti-PEG antibodies.^97^ As analyzed *in vivo* by Grenier *et al.*^98^ on PEGylated liposomes and polymeric NPs, using anti-PEG antibodies can have a significant neutralizing effect. Comparing the corona formed in naive mice, the exact impact of these antibodies on PC was found. The changes were analyzed according to the Ig deposited on the surface of NPs from the serum of PLGA–PEG NPs (poly(lactic-*co*-glycolic acid))-sensitized animals. This also alters the deposition of PC as apolipoproteins were found to be deposited on the surface of PLGA–PEG NPs compared to free methoxy–PEG chains and poly(lactic-*co*-glycolic acid) (PEG5k–PLGA). This might be relevant for nanomedicine given the implication of these proteins on the clearance of NPs in the bloodstream.

A solution was proposed by Wang *et al.*^99^ They suggested that adding α-glutamyl at the end of PEG should increase the circulating time of the polymeric micelles compared to bare PEG. Another challenge is the clearance of PEG–NPs as their accumulation in the spleen and the liver is significant. The work of Esposito *et al.*^100^ shows a method to overcome this issue. The *in vivo* biodistribution in mice was regulated by mixing amino-groups and PEG on the surface of polycaprolactone NPs. The accumulation of NPs in lungs, spleen and liver was investigated, lungs being the targeted organ containing cancer cells (B16F10 cells). This NH_2_/PEG coating was compared with PEG-coated NPs and with a human albumin layer (NH_2_/PEG–NPs@HA). The results indicate that NH_2_–NPs accumulate more in the lungs, than other NPs, as shown in Fig. 4. Additionally hemolysis calculated for all the NPs were less than 20% and showed no effect in *in vivo* studies.

### Precoating with proteins for *in vivo* controlled behaviors

**Fig. 5:**
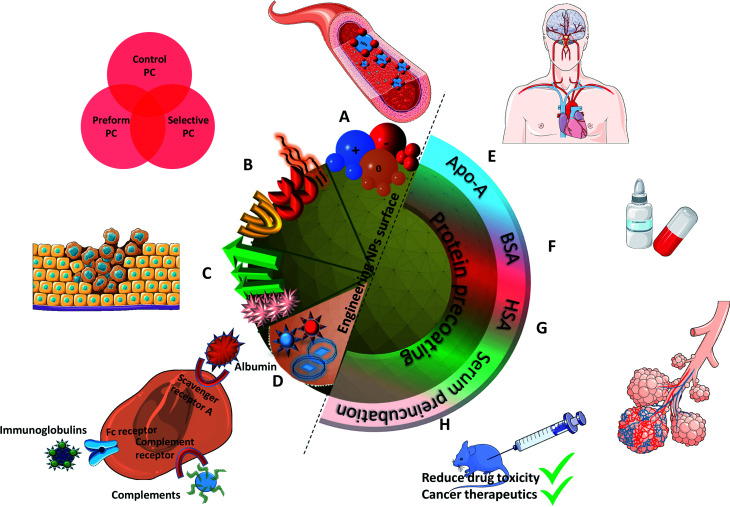
Summarized results of different controlled methods of PC formation: (A) negatively charged NPs result in longer circulation time causing greater accumulation, (B) different surface functionalizations improve biocompatibility, (C) protein specific functionalized groups result in tumor targeted drug delivery, (D) different types of NPs (liposomes, enveloped virus NPs) explore different binding receptors; protein precoating particularly with (E) (Apo-A) enables crossing the BBB, (F) BSA results in improving the oral bioavailability of drug, (G) HSA proteins help improving the air blood barrier as observed in *in vivo* lung lining, and (H) pre-incubating NPs with serum results in adsorbing more proteins that reduce the drug toxicity and help in cancer therapeutics.

Clinical applications of NPs would be limited if their surfaces would adsorb proteins in an uncontrollable and non-reproducible manner. Researchers came up with a promising pathway to prevent these non-specific approaches by developing a biohybrid precoating with a PC around the nanomaterials. Adding peptides or a protein coating, grafting aptamers, antibody and other molecules as already discussed in the previous section and summarized in Fig. 5 could further help in improving the efficacy of NPs in biomedical applications.^101^ For instance, crossing the blood brain barrier (BBB) is possible by binding Apo-E to the surface of polysorbate-coated NPs *in vivo* thereby facilitating the transport of bound dalargin or loperamide to the brain.^102,103^ Mahmoudi *et al.*^104^ also considered *in vivo* 3.5 nm SPIONs with different surface charges on dextran (NH_2_, COOH and unmodified) in a BBB mouse model. MRI observations on mice showed that unmodified and negatively charged NPs were present in the brain vessels 5 min after administration. They assigned this behavior to the presence of Apo-A1 protein in the corona of negatively charged NPs.

On the other hand, non-specific binding of proteins to NPs can lead to the cell clearance by macrophages through the MPS of the liver and spleen.^59^ Opsonins adsorption (fibrinogen, Immunoglobulin G (IgG), complement factor) were used to facilitate phagocytosis along with the elimination of NPs from the bloodstream.^105^ Conversely, on dysopsonin binding Human Serum Albumin (HSA), Apo facilitates prolonged circulation time in blood.^106^ Park *et al.*^107^ also demonstrated that their silica NPs show less macrophages uptakes and a reduced complement activation when coated with BSA, HSA, fibrinogen and complement factor H. IgG, on the other hand, was confirmed to increase macrophage uptakes.

Several studies also report that the formation of PC around NPs contributes to the loss of drug targeting. However, having a protein coating/PC evaluation prevents a non-essential binding of proteins as explored recently by Chung *et al.*^108^ who developed targeting-enhancing paclitaxel (TENPA) NPs where paclitaxel was encapsulated with a human serum albumin–haemin complex. They successfully showed that this hinders the formation of PC *in vivo* and that the structural stability was maintained enhancing the cancer targeting efficiency. These properties of TENPA lead to the accumulation of paclitaxel that was 4.1 times higher than that of nanoparticle albumin–bound paclitaxel (Abraxane®). This turns out to be an ideal drug in cancer therapeutics since the toxicity observed *in vitro* and *in vivo* was less than that of Abraxane and another formulation of free paclitaxel (Taxol®) in cancer bearing mice.

Another interesting approach was used to understand the mechanism of PC in lung lining fluid with its impact on the lung clearance in rats. As shown by Konduru *et al.*,^109^ coating albumin on gold NPs increases their uptake in macrophages suggesting that PC enables particle recognition, phagocytosis, and processing by alveolar macrophages as well as their translocation across the air blood barrier *in vivo* in lung lining fluid. Additional studies further supported the hypothesis that the formation of PC around NPs promotes its biological impact. Au–thiol–Fe_3_O_4_–SiO_2_–PEG^78^ showed more PC around them after incubation and after coating a PC (human plasma) resulting in increased cellular ATP levels and produced 
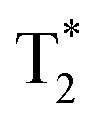
 contrast agents *in vivo*.

However, these artificial precoatings are not restricted to NPs to be injected *via* an i.v. route. Wang *et al.*^110^ worked on liposomes containing insulin for oral administration. They coated BSA (Bovine Serum Albumin) onto these liposomes to improve the oral bioavailability of insulin. More precisely, this coating enhances the ability of these NPs to penetrate the mucus faster and deeper. They observed after intrajejunal injections in diabetic rats (type 1) a better intestinal absorption and a significant hypoglycemic effect.

Gonzalez-Moragas *et al.*^111^ used a simple *in vivo* model (*Caenorhabditis elegans*) to analyze protein (BSA)-coated SPIONs. They reported changes in the toxicological and biodistribution profiles compared to citrate-modified SPIONs. BSA–SPIONs showed lower mortality than citrated SPIONs in a broader range of concentration proving that BSA has a protective role for nematodes as well. In another study,^112^ BSA–SPIONs were orally administered in the same model. The NPs were protected from acid degradation. They remained monodispersed in the lumen microenvironment and also prevented the direct contact of the inorganic core with the worm's body leading to a higher biocompatibility which was not present in the case of citrated SPIONs. This also helped explore a different pathway for some NPs entering in the nematode's enterocytes through endocytosis. Similarly, Peng *et al.*^113^ evaluated the NPs biostability *via* preformed PC using BSA. The drug release (coumarin 6) was found to be slower and the stability was enhanced in other organs and liver homogenate. On careful evaluation of NPs biodistribution on mice blood, it was observed that BSA-coated NPs were more stable in tissues, as the concentration of BSA-coated NPs was higher than for unmodified NPs in all the tissues at 15 min post-injection. Unmodified NPs were also metabolized or eliminated faster. As a result, preforming a PC (BSA) is beneficial in developing nanoformulations with stable drugs.

In all the preceding works, the pre-coating consists in putting specific chosen proteins, albumin in most cases. Yet a more complex but harder to control coating can be achieved by pre-incubating NPs into a biologically comparable media: serum or plasma.

Lin *et al.*^114^ studied PC on NPs incubated in different serum concentrations. They monitored the amount of Apo bound to NPs. Despite surface chemistry or morphological differences, preincubation at higher serum concentration leads, as might be expected, to a higher amount of proteins bond to NPs. It also confirmed that the functionalization by carboxylic acid resulted in a reduced protein adsorption.

**Fig. 6:**
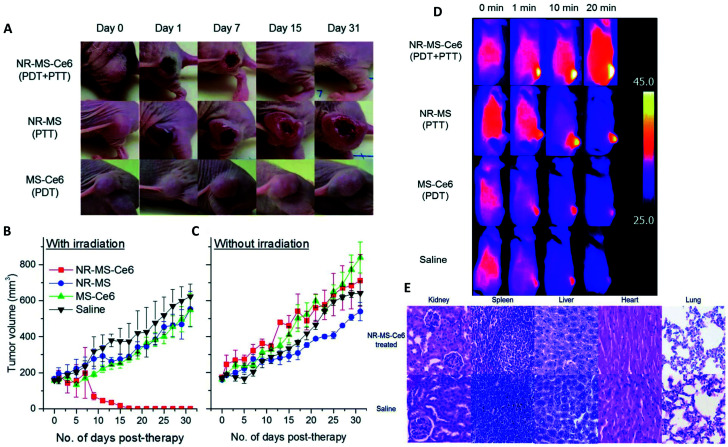
(A) Mice tumors observed when treated with NR–MS–Ce6 (10 mg kg^−1^ Au loaded with 9.63 μg kg^−1^ Ce6) (PDT + PTT), and an equivalent concentration of its control NR–MS (10 mg kg^−1^ Au) (PTT) and MS–Ce6 (9.63 μg kg^−1^ Ce6) (PDT). (B) Tumor volumes as a function of time following the i.v. delivery of NR–MS–Ce6 and related controls, and laser irradiation (*n* = 5). (C) Without laser irradiation, the localization of NR–MS–Ce6 did not cause any inflammatory response and a continuous tumor growth was observed over time, similar to the controls (*n* = 3). (D) Near infrared (NIR) thermal images showing tumor-localized irradiation of tumor-bearing nude mice and a rapid and localized PTT heating of the tumor within 1 min of irradiation. (E) Hematoxylin and eosin (H&E) staining of tissue sections from the major organs showing that the histological features between NR–MS–Ce6 (10 mg kg^−1^ Au loaded with 9.63 μg kg^−1^ Ce6) treated mice for PDT + PTT and saline-treated control mice were similar, with no abnormal phenotypic features observed. Figures reproduced from ref. 115 published by the Royal Society of Chemistry.

The advantage of preforming a PC was also successfully demonstrated prior to i.v. delivery. The time for Apo to bind a Au nanorod (NR) surface to form a PC was increased.^115^ These bound Apo in NR–MS–Ce6 (PC from Mouse Serum, MS and photosensitizer Chlorin e6, Ce6) act as endogenous targeting ligands to promote localization. This localization was observed 6 h post injection followed by a rapid rise in temperature localized in tumors. within 3 min of irradiation and compared to bare NRs. This showed a great potential for drug delivery (passive release of Ce6) and cancer treatment using PDT and PTT as can be seen in Fig. 6. Another surface coating was studied by Cai *et al.*^116^ using mouse serum albumin proteins prior to i.v. injection studied to analyze if preformed PC influence the *in vivo* NPs metabolic pattern and its toxicity. MS–Au NRs adsorbed more opsonin proteins resulting in an efficient liver-targeting. An increase by more than 80% of injected MS–Au NRs was observed in the liver within 24 h compared to unmodified NRs. The study also reveals that opsonin mediates the hepatic uptake of Au NRs. The resulting NPs were heat stable and due to the preincubation, they managed to escape phagocytosis by Kupffer cells and were found in hepatocytes.

Pre-adsorbed proteins control PC and also help in reducing the toxicity of the developed NPs. Recent works with both pyrogenic and colloidal silica NPs having a pre-adsorbed PC resulted in the suppression of the cytotoxicity and a greater cellular uptake inside A549 cells. Interestingly, RAW264.7 macrophages show a response similar to A549 cells, where precoated particles with PC induce the uptake and pro-inflammatory responses.^117^ Since NPs in the lungs do not limit themselves to epithelial cells but also encounter macrophages, it is essential to study the response of PC in macrophages as well. Therefore, PC turns out to be an effective tool in the lung tissue to control the toxicity of nanosilica particularly in pulmonary drug delivery. With a similar approach, pre-adsorbed PC not only facilitates the NPs behavior but can also help in reducing the drug toxicity by preincubating silica NPs in bovine serum. Giri *et al.*^118^ suggested it in a preliminary work to control the solubility and toxicity of testosterone, which engenders unwanted side effects if administrated alone. Indeed, it causes liver tumors when orally administrated, skin reactions when administrated *via* patches, or needs repeated and painful intramuscular injections.

For a better understanding and control of the *in vivo* biological behaviors of NPs, it is possible to tailor the PC either *via* an accurate surface functionalization or *via* precoatings before further use. Many studies demonstrated strong results in this way. They achieved improved blood circulation or targeting efficacy. However, questions remain regarding the PC formation directly *in vivo*.

## Studying PC *in vivo*: the way to improve nanomedicine?

In comparison to *in vitro* and fundamental studies, characterizations of the PC recovered after *in vivo* injections are still scarce. The first study focusing on *in vivo* analyses of PC was published in 2014.^119^ In this study, polymer-coated SPIONs with different charges were injected in rats' bloodstream and recovered a few minutes after injection to separate and analyze their PCs. The biological behaviors of SPIONs were compared to the proteins found. Negative and neutral NPs showed the same liver uptake delayed compared to positive NPs. This is explained by the lower proteins adsorption of positive coatings compared to neutral and negative ones during the *in vivo* evaluation. This behavior is quite different from what was observed *in vitro* and is strongly dependent on the composition of PC. The authors also compared PC *in vivo* to *in vitro* experiments on rat's plasma. Very different proteins were adsorbed on the surface of SPIONs. They concluded on the difficulty to compare *in vitro* and *in vivo* analyses and the unpredictability of PC *in vivo* because it results from a dynamic process. Since then, therapeutic NPs have been extensively explored^120^ but few papers are focusing on the studies of the *in vivo* interactions of nanohybrids with proteins (less than 20 at the date of the writing).

### Parameters influencing the PC *in vivo*

**Fig. 7:**
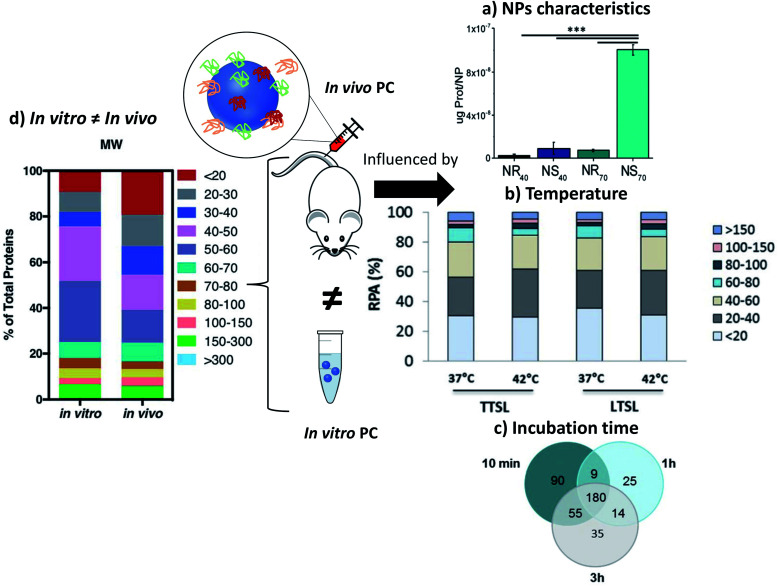
*In vivo* PC is influenced by NPs characteristics such as (a) sizes and morphologies. Concentrations of proteins found *in vivo* on gold nanorods (NR) and nanostars (NS) of 40 and 70 nm reproduced from ref. 126 with permission from the Royal Society of Chemistry; external factors such as (b) temperature influencing the formation of *ex vivo* PC (ordered in molecular weights) on temperature-sensitive liposomes encapsulated with traditional (TTSL) or lysolipid-doxorubicin (LTSL) reproduced from the work published in ref. 123 Copyright© (2018) with permission from Elsevier and (c) Venn diagrams report the number of unique proteins identified in the *in vivo* corona formed on liposomes 10 min, 1 h and 3 h post-injection and their respective overlap. Reproduced from ref. 129 – published by the Royal Society of Chemistry. *In vivo* protein corona (PC) is always different than *in vitro* protein corona (d) percentage of proteins found *in vitro* and *in vivo* on lipidic NPs classified according to their molecular weight reproduced from ref. 140 published by the Royal Society of Chemistry.

Some studies report the influence of environment and the characteristics of NPs on the adsorption of proteins. As shown above with different surface charges,^119^ the chemistry of the surface coatings leads to different proteins adsorption. Varying the peptides on the surface of polystyrene NPs^121^ and the liposomes or leukosomes (biomimetic liposomes) chemistry^122,123^ was shown to influence the nature of the adsorbed proteins. For PEGylated stealth liposomes,^124^ it was also demonstrated that 1 h after tail vein injection in mice PC did not cover the whole surface of NPs. In fact, 0.5 to 1.9% of surface covering was observed for non-targeted liposomes (without antibody) and 0.6 to 2.7% for targeted liposomes (with antibody). Serum albumin came out to be dominant proteins in the corona of the liposomes, which probably prevented it from compromising the functionalization with antibody trastuzumab. The density of polymers also affects PC. For example, increasing the PEG density decreased the adsorption of Apo leading to a longer residential time.^125^ The size and shape of NPs are also crucial parameters influencing PC *in vivo*. For example, gold NPs and nanostars^126^ size and shape modified the amount of adsorbed proteins (Fig. 7a). The NPs with the highest specific surface area (the biggest gold nanostars) adsorbed more proteins than the other NPs. However, the complexity of the protein layer found on the surface of these NPs was not related to these characteristics. The bio-circulation and the spleen and hepatic uptakes were inversely proportional to the size of NPs. A kinetic study with different sizes of Au NPs of the *in vivo* PC formation and their biodistributions was coupled to computer models helping to predict the biological fate of NPs^127^*. In vivo* studies generate a lot of data and using predictive systems seems to be one of the solutions to better anticipate the potential *in vivo* behavior of NPs.

External parameters also influence the *in vivo* PC. Liu *et al.*^128^ studied the influence of static magnetic fields and demonstrated that this external force modifies the nature and quantity of adsorbed proteins on magnetic NPs. Adding a magnetic field during incubation in serum *in vitro* did not modify PC significantly. However, *in vivo* the number and nature of the adsorbed proteins was increased by 25% when a magnetic field was applied. Further exploration of the effect of the magnetic field on magnetic NPs in order to control PC would be of great interest particularly in nanomedicine. In the presence of a magnetic field, the amount of adsorbed protein increases in particular in the HC layer. It is therefore an effective way to reduce the unexpected adsorption of protein by analyzing the time of interaction.

Temperature can also be an important parameter influencing the PC formation *in vivo*.^123^ Two different thermosensitive phospholipids were injected in mice and then recovered from the mice blood stream. The adsorption of proteins was modified by changing the temperature of *ex vivo* incubation from 37 °C to 42 °C. While this study did not investigate the *in vivo* influence of temperature, this approach suggests that even a slight change of temperature can affect PC as well as the associated nano-thermotherapy (Fig. 7b).

In other studies, Hadjidemetriou *et al.*^129^ and Corbo *et al.*^122^ studied composition evolution of PC *in vivo* around liposomes injected in mice as a function of time (Fig. 7c). In the first study, the authors demonstrated that the amount of adsorbed proteins on liposomes did not change significantly with the time of sampling. However, the nature of proteins was drastically different demonstrating a dynamic process of protein adsorption due to the flow of the bloodstream. The nature of proteins did not really influence the physicochemical characteristics of NPs (hydrodynamic sizes and zeta potentials). In the other study,^122^ the *in vivo* PC composition changed drastically between 10 min and 1 h post injection. The number of different proteins increased by 33% after 1 h of *in vivo* incubation compared to 10 min. The nature of proteins found on the NPs' surface changed too. Most of the proteins found 1 h after incubation have a coagulation function demonstrating the dynamic evolution of the *in vivo* PC which could lead to a total change of the behavior of NPs. Chen *et al.*^130^ studied how fast PC can change after injection of magnetic nanoworms. The authors studied the dynamic behaviors of the PC formation on different protein-precoated dextran-stabilized nanohybrids 5 min after injection. They focused their study on the complement component 3 (C3) which is one of the most abundant proteins *in vivo*. They demonstrated that a competition exists in proteins adsorption even for proteins known to bind specifically and easily on NPs once injected.

Another external factor influencing the *in vivo* PC is the health of the injected patient. In another study^131^ from Hadjidemetriou *et al.*, liposomes were injected in healthy and tumor-grafted mice. The health of animals influenced the amount of PC and the proteins found in the bloodstream and on the NPs. The concentration of recovered liposomes, as calculated by Stewart assay, was found to be ∼30% of the injected dose. The authors demonstrated that their NPs were able to target proteins excreted by tumors and used them as tumor's biomarkers trackers. This assumption was also tested *in vivo* on human ovarian carcinoma patients.^132^ For the first time, the PC of liposomes injected in human was analyzed after blood collection. This preliminary promising study characterized the physicochemical evolutions of liposomes after injection and their ability to adsorb proteins from the human blood stream. As observed in previous studies, the hydrodynamic sizes and zeta potential of liposomes did not change. NPs were able to adsorb proteins overexpressed in this cancer helping their *ex vivo* detection and proving the potential of these liposomes as cancer biomarkers tracers. Such a study demonstrates the interest of studying PC interactions on NPs after injection. It is to note that lipoproteins and liposomes might have similar lipid compositions. Liposomes are typically separated from other biological compounds with techniques varying from one team to another, such as ultracentrifugation coupled with membrane ultrafiltration.^133^ Another example is the chromatographic separation, specifically fast protein liquid chromatography, which enables the separation of liposomes from lipoproteins and plasma components.^134^

Several studies analyzing the formation of the *in vivo* PC led to the same conclusion as *in vitro*. Interactions between NPs and proteins are influenced by external (environmental) factors which are difficult to control and also by the NPs' surface chemistry that can be engineered.

### *In vivo* analysis of PC: *in vitro* and *in vivo* measurements are not related

The key factor for the failure of clinical translation of nanotherapeutics in nanomedicine is the poor link between the *in vitro* “assessments” and the *in vivo* “outcomes”. Some studies illustrate the strong differences between the *in vitro* and *in vivo* results. Among them, one recurring issue is PC, in particular its screening effect hindering the interactions between NPs and their targets.^135,136^ Hadjidemetriou and co-workers studied the *in vivo* behavior of PC on liposomal NPs. They demonstrated that *in vitro* and *in vivo* incubations of their liposomes led to different PCs (Fig. 7d) which were also influenced by their coatings (PEG) and functionalization with antibodies.^137^ The formation of a PC was evaluated on liposomes *in vivo* after tail vein injection into CD-1 mice. Liposomes were recovered 10 min post injection. Plasma from CD-1 mice was used *in vitro* to mimic the *in vivo* condition. The mass of the proteins adsorbed on pegylated-liposomes was lower than that for bare liposomes after *in vitro* or *in vivo* incubations leading to a lower cell internalization (MCF7 and C33a cells). The antibody-functionalized NPs adsorbed less proteins after *in vitro* incubation compared to *in vivo* incubation. These nanohybrids kept their targeting capabilities for cells after incubation. Additionally, lipid composition of liposomes is also a key factor in shaping the PC *in vivo* once introduced in the medium as explained by Storm *et al.*^138^ where they compared the liposomes degradation dependent on the lipid composition when taken up by liver and spleen on intravenously administered of [^3^H]inulin-labeled vesicles to tumor-bearing animal.

Other studies have also concluded on the importance of focusing on *in vivo* characterizations of PC as *in vitro* measurements have usually given different results whatever the nature of particles. As an example, the PC of polystyrene NPs^121^ with different coatings were analyzed 10 min after injection in mice and compared to the PCs found after incubation in mice serum the authors found significantly more albumin but also clusterin capable to reduce the nonspecific uptake more on the *in vivo* PC than on the *in vitro* PC. Fibrinogens, which are responsible for PC aggregation, were more abundant *in vitro* than *in vivo.* Possible reasons for different concentrations of fibrinogen in various biological medium might lie in the preparation step which depletes the serum of coagulating factors such as fibrinogen and lowers the protein concentration.^121^ Furthermore, in another study^139^ from Kottana *et al.*, effects in the change of conformation of fibrinogen instead of abundance were investigated. It was demonstrated that *ex vivo* adsorption of fibrinogen on positively charged PVA-coated SPIONs' affected the conformation of this protein itself resulting in platelets activation but not aggregation. The effect of protein conformation in PC is an interesting point to focus on to understand the impact of PC in the potential change of activities of the adsorbed proteins. Back to the abundance of proteins, Amici *et al.*^140^ also showed that the abundance and composition of PC is different *in vitro* (FVB/N mouse plasma, Friend Virus B NIH Jackson, inbred mouse strain) and *in vivo* (FVB/N mice) in the case of liposomes. A larger variety of proteins was found *in vivo* (500) compared to *in vitro* (267). The same conclusions were obtained for inorganic NPs such as SPIONs.^128^ For SPIONs functionalized with glutamine, the mass and nature of the proteins found on the SPIONs' surface were totally different. 669 proteins were found *in vivo* after injection in mouse when only 100 different proteins were found *in vitro* after serum incubation. Furthermore, only 56 proteins were common to both cases. Such results demonstrate that understanding PC for *in vivo* applications is difficult and maybe even impossible when using *in vitro* experiments. In addition to the differences observed in the number or amount of proteins adsorbed *in vitro* and *in vivo*, thermal-triggered drug release too cannot be simply analyzed by *in vitro* incubation. When temperature sensitive liposomes (TSL) were studied, slight variations were observed for their drug release profile *in vitro* and *in vivo*. The performance of TSL *in vitro* fails to predict the *in vivo* behavior directly.^123^ The differences in the structural configuration and composition of the formed PC in both conditions mainly results in a faster release profile *in vitro* than *in vivo*. For example, incubation of superparamagnetic NPs *in vitro* with different protein solutions at various temperatures provides several degrees of protein coverage and therefore numerous PC compositions which thus define drug release.^141^

## Discussion

Authors observe and try to understand the influence of the NPs' surface chemistry (coating, size, shape, charge, precoating) as well as external parameters on *in vivo* proteins adsorptions in order to control their biological behaviors. A deep reflection on PC *in vitro* seems to be one of the key factors for the further development and comprehension of the *in vivo* nanomedicine.^142^ Some papers agree with this postulate and to study in details the formation of PC *in vivo*. Recent analyses on this *in vivo* PC have brought interesting results but have also raised many discussions and open questions. This is mainly due to the complexity of the studied models as shown by the main results that pointed out important differences between *in vitro* and *in vivo* observations.

First, it was shown that surface engineering and in-depth analyses bring interesting results on PC's understanding. Studies also clearly demonstrated that classic *in vitro* analyses or modifications will not help to fully understand, predict, and simplify a complex system such as the *in vivo* one. However, *in vitro* still should not be put aside but could be used as a tool for the understanding: besides an *in vitro* followed by an *in vivo* analysis and a comparison between those two, one can also deeply study *in vitro* a specific protein found *in vivo* in order to gain more insight into its molecular mechanisms. In this case, *in vitro* offers a fully easy controllable environment for NPs-specific protein interactions. Thus, *in vitro* and *in vivo* are equally important to understand the interactions.

Furthermore, many parameters from the system itself are still very hard to simulate^143^ even if they influence the amount and the concentration of proteins in biological fluids. For example, *in vitro* analyses are usually performed in serum when the same *in vivo* experiments are performed in plasma. Differences of biological behaviors were observed on silica NPs after incubation in human serum and plasma:^144^ NPs were more internalized by macrophages in plasma than in serum. The authors concluded that the higher amount of fibrinogens and opsonins in plasma compared to serum affect the phagocytosis of NPs. Working on plasma instead of serum might be a first step to help predict the *in vivo* behavior from *in vitro* experiments. It is also interesting to keep in mind that in the case of blood circulation, clearance of NPs can change the population of NPs and select artificially the PC of more circulating NPs. Then the average quantification of *in vitro* PC on a complete population of NPs compared to *in vivo* PC on a sub-population of NPs may differ drastically. Another reason for differing results between *in vitro* and *in vivo* PC may also come from the design of the experiments and the choice of the *in vitro* serum. Most studies usually analyze the *in vivo* PC on each animal^119,140^ but compare it to a serum pooled from many animals. The concentrations, amounts and nature of the proteins may statistically differ and lead to significant altered results. Correctly designing the experiments with enough data might be a solution to improve the comparison (see below).

The health of the patients also strongly influences the proteins composition in the PC. Hajipour *et al.*^145^ studied the incubation of graphene NPs in the blood of human patients presenting various diseases leading to different “personalized” PC exhibiting different biological behaviors (cytotoxicity, inflammation responses *etc.*). Additionally, hemolysis was observed in various diseased patients. Diabetes and thalassemia showed a hemolytic activity higher than blood cancer patients. This difference affects PC formation due to plasma alteration. This can be due to the autoimmune hemolytic anemia, which occurred during the different diseases and influences the competitive binding of proteins on the NPs surface.

**Fig. 8:**
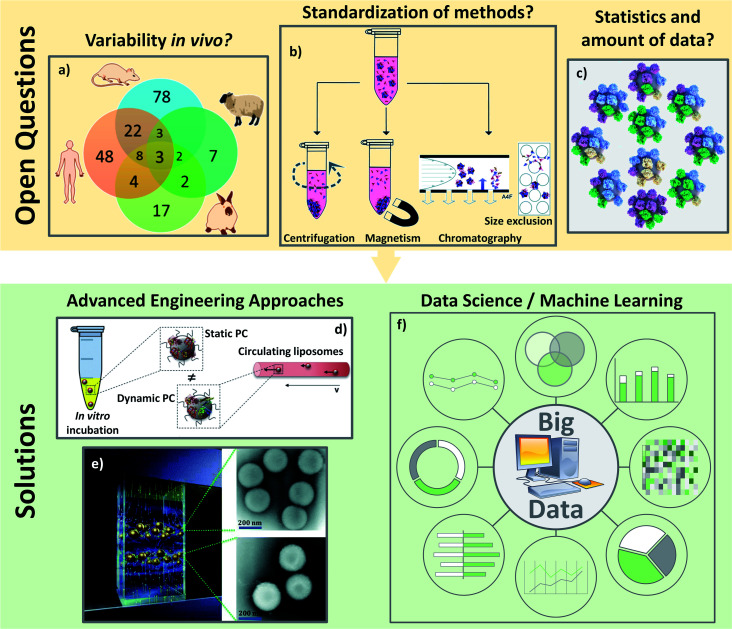
Open questions about the PC analyses *in vivo*: (A) there are a lot of varying biological parameters *in vivo* such as different PC coronas found on polystyrene NPs incubated in different animal serum as represented on the Venn diagram: adapted with permission from ref. 147. Copyright© (2018) American Chemical Society; (B) characterizations of PC are not yet standardized such as the separation techniques of the hard PC leading to difficult comparisons: from ref. 153 – published by the Royal Society of Chemistry; (C) average analyses of PC are also not reliable. There is no average PC but a multitude of different PC around various different NPs: reproduced from the work published in ref. 157 Copyright© (2016) with permission form Elsevier. Solutions for the analyses of PC especially *in vivo* could come: from engineering development to standardize and mimic the biological environment like: (D) microfluidic to incubate NPs with proteins in dynamic environment: adapted from the work published in ref. 160 Copyright© (2016) with permission from Elsevier or (E) magnetic levitation method separating NPs depending on their amount of PC to get closer to the “real” PC: reproduced from ref. 162 with permission from the Royal Society of Chemistry and/or (F) from data science to pool and analyze all the results leading to data smoothing and a global understanding.

The concentrations and types of plasma proteins also differ from the animal model used. Intuitively, the PC formed is different from one to another,^146^ thereby resulting in different biological behaviors. This implies a complex extrapolation from animal to human^147^ (Fig. 8a). It raises another issue as most *in vivo* reports deal with the mice model, and only very few analyze both simultaneously. Solorio-Rodríguez *et al.* explained the clear difference between human and mouse plasma PC profiles in an active therapy model using SiO_2_.^148^ This explained very well the limitation of *in vivo* models translated to clinical use. Thus, the PC profile must be considered in the interpretation of preclinical trials when developing efficient and safe nanomedicines. Biological fluids are also regulated systems with many varying physical parameters such as temperature, pH as well as the composition and concentration of proteins. Other differences were also observed depending on the studied biological fluids. However, all of the publications studying PC *in vivo* rely on i.v. administration. Some authors discussed the importance of the fluids in which NPs are injected. It was demonstrated that the PC of NPs differs when incubated in blood or lymph.^149^ Future *in vivo* experiments will have to study the PC formation using other injection routes (intramuscular, inhalation, ingestion *etc.*). Additionally, the preparation of blood samples may also interfere with PC selection and analysis. In the studies described above, different anticoagulant agents which were used to collect blood (EDTA,^124^ heparin^121^ …) may also affect protein interaction. Heparin, for example, will neutralize thrombin, the protease responsible of the formation of fibrin from fibrinogen. EDTA, on the other hand, is a chelating agent which will form a complex with the calcium ions. Such differences in the anticoagulation mechanisms has to be kept in mind since they are likely to induce slight changes in the composition of plasma samples, possibly resulting in a different PC formed on the NPs. Planning correctly *in vivo* experiments, especially while analyzing PC, remains a challenge. It would be important to set up some standardized procedures such as: (i) using the same anticoagulant; (ii) enough animals for statistics or (iii) using “our own *in vitro* serum” from the animals studied. And even if it might prove very complicated, it would also be useful to control some biological parameters such as the health or alimentation of the animals. A possible solution to optimize the understanding of *in vivo* PC might come from the use of genetically modified animal with under-expression of some proteins to study, for example, their potential influence on NPs biocirculation.

In addition to experimental set-up, PC characterizations are still complicated to perform and hardly reliable due to the many parameters depending on the isolation methods and the analyses of the results. While some protocols exist to explain how to separate and isolate the “real” PC,^150,151^ recent studies are still highlighting the lack of standardization in the PC analyses especially regarding sample preparation^152^ in the separation protocols^153^ (Fig. 8b). The most used separation technique is centrifugation which allows the characterizations of proteins at the surface on many kinds of NPs including very small ones.^153,154^ Nevertheless, when other methods are available as in the case of PC on SPIONs, Bonvin *et al.*^155^ demonstrated that the proteins found after centrifugation and after magnetic separation are different. They concluded that only 50% of the PCs found for both isolation methods could be considered as the “real” PC. This problem of finding a proper method to characterize PC is a common problem for *in vitro* and *in vivo* studies. For instance, how can one be sure that the proteins analyzed are really the proteins present on NPs or the result of protein isolation failure? Moreover, analyses of PC usually focus on the effect of one parameter after another. The question arises whether such observations still hold true in general.^15^ The dependence of biological behaviors on one particular physicochemical parameter or on a combination of them (pH, temperature, concentrations of proteins *etc.*) suggests that the limited model discoveries in literature are not enough to be extrapolated in a straightforward way to *in vivo* behaviors. Last but not least, one issue regarding PC analyses is related to the massive amount of data obtained with the dedicated characterization techniques. Even if the methods of isolation were to become standardized, the proteins/nanoparticles interactions will always need statistical analyses. Galmarini *et al.*^156^ demonstrated the crucial importance of replicates and statistics in the analyses of proteins adsorbed *in vitro* onto silica NPs' surfaces. In this study, they discussed the fact that there are few papers running control experiments when analyzing PC. With the established conditions, they found for instance that albumin and Apo (two of the most abundant proteins in serum) were more present on the analysis device than on NPs. It is then important to avoid biased results due to the lack of a proper set up of experiments especially *in vivo* where there are already many biological parameters. They also proved, by running their experiments in triplicate, that more than 60% (out of the 250 proteins found) were not significantly present leading to a fundamental need of statistical approaches to analyze the different proteins. The absence of systematic control experiments or replicates can be explained by the amount of data coming from the experiments. It is not unusual to obtain several hundred proteins in a PC study and the classification of the data as well as the data processing can be an issue. Thus, the importance of proper set-up, repetition and reproducibility of isolation and analysis methods becomes crucial to analyze the *in vivo* protein interactions with NPs. A last issue for data analyses is that PC is a statistical phenomenon. Forest and Pourchez^157^ already noted that since proteins and NPs have approximately similar sizes, an average approach of PC is not very reliable. Most techniques usually analyze hundreds of different proteins. It is not possible for all these proteins to stick at the surface of a single nanoparticle. Therefore, considering an average PC may not be trustworthy because no NP has this exact average composition (Fig. 8c). No analytical solution currently exists as it is impossible, especially *in vivo*, to analyze the PC on single NPs.

All these unaddressed concerns are particularly challenging for the future of PC studies *in vivo*. The solution may come from new isolation and analysis techniques. As *in vivo* conditions are, for the moment, difficult to compare to those *in vitro*, mastering the analyses is required to be able to understand and control the adsorption of proteins on NPs once injected. Some experiments have highlighted that the dynamic process of the PC formation is influenced by (i) the time of incubation and (ii) the flow of bloodstream. Since the PC formation in blood is a dynamic process, a better *in vitro* model may come from the use of microfluidic setups.^158^ Kari *et al.*^159^ and Palchetti *et al.*^160^ (Fig. 8d) suggest that dynamic *in vitro* analyses result in a different PC on NPs than static approaches. They claimed that the circulation of NPs *in vitro* might mimic the effect of bloodstream. Weiss *et al.*^161^ went one step further while studying the temporal evolution of PC with a dynamic setup. They found three different steps of protein interactions on silica NPs' surface. During the initial step the first proteins adsorbed in an irreversible manner directly onto the particle surface. In the second step, the corona interacts irreversibly with other proteins forming an intermediate layer where the PC does not directly adsorbed to the surface but belongs to the inner HC. They demonstrated that HC is actually a double layer of proteins strongly interacting with the NPs' surface and with strongly adsorbed proteins. During the last step, circulating proteins form the outer SC layer by reversibly binding with proteins from the HC. An innovative magnetic separation technique (Fig. 8e) developed by Ashkarran *et al.*^162^ seems to be a good alternative to centrifugation to isolate PC. This method relies on magnetic levitation to prevent proteins from sticking to the surface of the isolation device. This technique leads to a more accurate determination of the composition of PC by separating NPs by the quantity of proteins they have on their surface preventing in part the “average analysis” discussed above. To analyze PC *in vivo*, a prominent work from Bargheer *et al.*^163^ used radiolabeling as a promising tool to probe the fate of an artificial PC. To do so, they tracked transferrin or albumin labelled with ^125^I, allowing to study the uptake and degradation. Despite this solution being restricted to only a few proteins, labelling PC could clearly help understand its *in vivo* behavior. Such engineering techniques should be kept in mind to bring back together the *in vitro* and *in vivo* behaviors of PC.

Accurate and standardized procedures were and will be proposed^151^ and will help to better compare the data from PC. However, it is now accepted that the massive number of varying parameters in biology and in analytical science might be too important to be controlled. Despite the fact that modeling such complex molecules with computational simulation^164–166^ and machine learning^167^ is still a very laborious task, this discipline would clearly benefit in the years to come from a fundamental understanding of the parameters responsible of the PC formation at the NPs' surface. In the meantime, data management with advanced engineering approaches may help to have them sorted and to analyze the PC formation to smooth the bias due to the operator, methodology or the studied model (Fig. 8f).

## Conclusions

Starting from what is known of the PC formation; many studies are trying to tune *in vitro* the NP surfaces with chemical modifications or directly with protein coatings in order to improve the *in vivo* biological behaviors of their nano-tools. In such studies, controlling the NP's material parameters may help controlling their biomedical behaviors. In other studies, the NPs are put *in vivo*, their PC is characterized and it seems to obey the same rules as *in vitro* (same parameters influencing protein adsorption). However, comparative studies between *in vitro* and *in vivo* PC have shown very different results. These differences are linked to the various parameters whose control still need to be improved in order to better optimize the *in vivo* PC: (i) set-up, reproducibility and repeatability of experimental and analysis method; (ii) accurate statistics on the obtained data; (iii) reliability between *in vitro* analyses and *in vivo* “real” behavior and last but not least, (iv) the control of the external physicochemical and biological parameters influencing PC *in vivo*. There is still room to analyze, compare and propose many mechanisms of protein interactions in order to control the NPs' behavior but the complexity of the systems might slow down the full understanding of the *in vivo* PC. It is equally important to evaluate all the experimental conditions from the selection of the right *in vivo* medium to a careful choice of the analysis models in order to predict the nanosystem's biological responses. With each altered parameters, the adsorbed proteins are different *in vitro*, *in vivo* and in human samples, which influences functionality of nanomaterials. However, some promising studies are proposing another approach, *in vivo* protein fishing, to be used as a diagnostic tool. Instead of trying to fully understand the *in vivo* behaviors, NPs are used to interact with specific proteins and then they are removed from the organism for early diagnosis.^132,168^ With all the knowledge already acquired, it is clear that the control of the materials parameters will not allow for a full optimization of *in vivo* PC and therefore of the NP biological behavior without a full understanding of this topic that could be the next step in the development of PC-based personalized nanomedicine.

## Authors contribution

L. M. suggested this work. N. S., C. M. and L. M. prepared the original draft and J. B, N. M. and L. S. reviewed it.

## Conflicts of interest

There are no conflicts to declare.

## Supplementary Material
